# Optimization of Anodized-Aluminum Pressure-Sensitive Paint by Controlling Luminophore Concentration

**DOI:** 10.3390/s100706836

**Published:** 2010-07-16

**Authors:** Hirotaka Sakaue, Keiko Ishii

**Affiliations:** Aerodynamic Research and Development Directorate, Japan Aerospace Exploration Agency/Chofu, Tokyo 182-8522, Japan; E-Mail: kekoi@chofu.jaxa.jp

**Keywords:** anodized aluminum, pressure-sensitive paint, luminophore concentration, pressure sensitivity, temperature dependency

## Abstract

Anodized-aluminum pressure-sensitive paint (AA-PSP) has been used as a global pressure sensor for unsteady flow measurements. We use a dipping deposition method to apply a luminophore on a porous anodized-aluminum surface, controlling the luminophore concentration of the dipping method to optimize AA-PSP characteristics. The concentration is varied from 0.001 to 10 mM. Characterizations include the pressure sensitivity, the temperature dependency, and the signal level. The pressure sensitivity shows around 60 % at a lower concentration up to 0.1 mM. Above this concentration, the sensitivity reduces to a half. The temperature dependency becomes more than a half by setting the luminophore concentration from 0.001 to 10 mM. There is 3.6-fold change in the signal level by varying the concentration. To discuss an optimum concentration, a weight coefficient is introduced. We can arbitrarily change the coefficients to create an optimized AA-PSP for our sensing purposes.

## 1. Introduction

**Figure 1.**
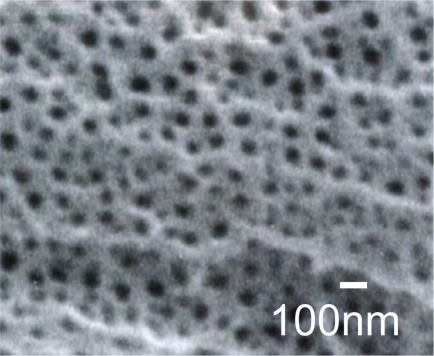
Nano-open structure of anodized-aluminum surface. Surface image was taken using a scanning electron microscope.

In aerospace engineering, anodized-aluminum pressure-sensitive paint (AA-PSP) has been used in unsteady flow measurements [1]. Because of its nano-open structure (Figure 1), AA-PSP yields high mass diffusion that results in a pressure response time on the order of 10 μs [2]. By applying an AA-PSP, we can obtain global surface pressure information instead of pointwise information that may result in wide applications in pressure detection fields.

**Figure 2.**
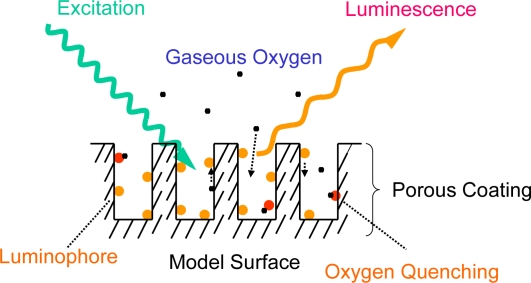
Schematic description of anodized-aluminum pressure-sensitive paint (AA-PSP).

An AA-PSP consists of a molecular pressure probe of a luminophore and an anodized aluminum supporting matrix. As schematically shown in Figure 2, the luminophore on the anodized-aluminum surface is excited by an illumination source and gives off luminescence. This luminescence is related to gaseous oxygen in a test gas, a process called oxygen quenching. Because the gaseous oxygen can be described as a partial pressure of oxygen as well as a static pressure, the luminescence from an AA-PSP can be described as a static pressure. See Section 2.3 for a detailed description.

**Figure 3.**
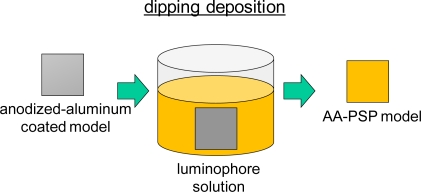
Schematic description of dipping deposition method.

The luminophore is applied on the anodized-aluminum surface by the dipping deposition method [3]. This method requires a luminophore, a solvent, and an anodized-aluminum coating. The application procedure is schematically shown in Figure 3. The anodized coating is dipped in the luminophore solution to apply the luminophore on the coating surface. Sakaue reported solvent effects on the characteristics of AA-PSPs [3]. When varying the solvent, he found that dichloromethane gives the best pressure sensitivity and signal level. On the other hand, the effect on the response time was minimal; it was within the uncertainty of the AA-PSP thickness measurement. The luminophore concentration may influence the AA-PSP characteristics, because the amount of luminophore on an anodized-aluminum surface may change with the concentration used in the dipping deposition, but the effects of this parameter on AA-PSP have not been studied. By following the present results, combined with the solvent dependency, various luminophores could be optimized for application on the anodized-aluminum surface. Those resultant AA-PSPs can be applied to global measurements in the fields of fluid dynamics, biology, and environmental science. In this paper, we describe the effect of the luminophore concentration in the dipping deposition method on AA-PSP characteristics. Steady-state characterizations are the focus of the present study, because an unsteady-state characterization of response time was minimally related to the dipping deposition method [3]. Characterizations include the pressure sensitivity, the temperature dependency, and the signal level. Because PSP in general has a temperature dependency, its relationship to the luminophore concentration was included [4].

## 2. Experiment

### 2.1. Sample Conditions

We chose bathophen ruthenium from GFS Chemicals as a luminophore, which is a conventional luminophore for AA-PSP. Based on Sakaue’s results [3], dichloromethane was chosen as a solvent. First an anodized-aluminum coating was prepared, followed by Sakaue’s procedure [3]. The coating thickness was 10 ± 1 μm as measured by an eddy current apparatus (Kett, LZ-330). AA-PSP samples were 10 × 10 mm in dimensions. The anodized-aluminum coating was dipped in the luminophore solution at room conditions for one hour. The concentration has the range of the fifth order of magnitude; it was varied from 0.001 to 10 mM. Table 1 lists the luminophore concentration conditions.

Prepared AA-PSPs were labeled (also listed in Table 1 as Sample ID). For each dipping condition, three samples were prepared to study the repeatability of AA-PSP preparation by the dipping deposition method. A total of fifteen samples were prepared.

### 2.2. Calibration System

**Figure 4.**
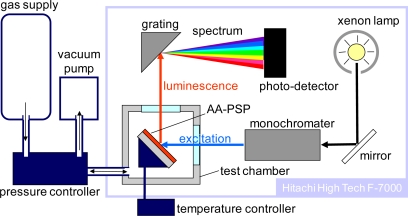
Schematic of AA-PSP calibration setup.

Figure 4 schematically describes the calibration system, which consists of a spectrometer (Hitachi High Technologies, F-7000) and a pressure- and temperature-controlled chamber.

This system characterizes the luminescent spectrum of an AA-PSP sample with varying pressures and temperatures. For characterization, an AA-PSP sample was placed in the test chamber. The excitation wavelength was set at 460 nm by a monochromator via a xenon lamp illumination in the spectrometer unit. The chamber has optical windows that passed the excitation from the illumination unit and the luminescence from the sample. The luminescence from AA-PSP samples was measured from 570 to 800 nm for a given pressure and a given temperature. The luminescent signal of an AA-PSP was then determined by integrating the spectrum from 600 to 700 nm. For pressure calibration, the chamber was connected to a pressure controlling unit (Druck DPI515), with settings from 5 to 120 kPa at a constant temperature at 25 °C. For temperature calibration, a sample heater/cooler was controlled to vary the temperature from 10 to 50 °C with a constant pressure at 100 kPa. The test gas was dry air. For the signal level characterization, all the AA-PSP samples were measured with the same optical setup in the spectrometer but replacing samples in the chamber at constant pressure and temperature of 100 kPa and 25 °C, respectively. Throughout our characterizations, reference conditions are 100 kPa and 25 °C. The pressure sensitivity, *σ*, temperature dependency, *δ*, and the signal level, *η*, were characterized from the luminescent signals of AA-PSPs. Definitions and procedures to derive these characterizations are described in sections 2.3, 2.4 and 2.5.

### 2.3. Pressure Sensitivity

Based on the Stern-Volmer relationship, the luminescent intensity, *I*, is related to a quencher [5]:

(1) $$\frac{I_{0}}{I}=1+K_{q}[O_{2}]$$

where *I_0_* is the luminescent intensity without quencher and *K_q_* is the Stern-Volmer quenching constant. The quencher is oxygen, which is described by the oxygen concentration, [*O_2_*]. For AA-PSP, [*O_2_*] can be described by the adsorption and surface diffusion of the adsorbed oxygen on an anodized-aluminum surface. We can describe [*O_2_*] by the partial pressures of oxygen as well as the static pressures. These are combined with Equation (1) to give the adsorption-controlled model [4]:

(2) $$\frac{I_{\mathrm{ref}}}{I}=A+B{\left(\frac{p}{p_{\mathrm{ref}}}\right)}^{\gamma }$$

where *A*, *B*, and *γ* are calibration constants, respectively. Here, *ref* denotes our reference conditions.

Pressure sensitivity, *σ* (%), describes the change in the luminescent signal over a given pressure change. This corresponds to a slope of the Equation (2) at the reference conditions:

(3) $$\sigma ={\frac{d(I_{\mathrm{ref}}/I)}{d(p/p_{\mathrm{ref}})}|}_{p=p_{\mathrm{ref}}}=B\cdot \gamma (\%)$$

To discuss the effects of *σ* on the luminophore concentration, it is non-dimensionalized as follows:

(4) $$\mathrm{norm}\sigma =\frac{\sigma -\sigma_{\text{min}}}{\sigma_{\text{max}}-\sigma_{\text{min}}}$$

where *σ*_max_ and *σ*_min_ are the maximum and the minimum pressure sensitivities, respectively.

### 2.4. Temperature Dependency

AA-PSP, like PSP in general, has a temperature dependency [4]. This influences the luminescent signal, which can be described as the third order polynomial in Equation (5):

(5) $$\frac{I}{I_{\mathrm{ref}}}=c_{T0}+c_{T1}T+c_{T2}T^{2}+c_{T3}T^{3}$$

where *c_T0_*, *c_T1_*, *c_T2_*, and *c_T3_* are calibration constants, respectively. We defined the temperature dependency, *δ*, which is a slope of the temperature calibration at the reference conditions [Equation (6)]. If the absolute value of *δ* is large, it tells us that the change in luminescent signal over a given temperature change is also large. This is unfavorable condition as a pressure sensor. On the contrary, zero *δ* means that AA-PSP is not temperature dependent:

(6) $${\delta =\frac{d(I/I_{\mathrm{ref}})}{\mathrm{dT}}|}_{T=T_{\mathrm{ref}}}=c_{T1}+2c_{T2}T_{\mathrm{ref}}+3c_{T3}T_{\mathrm{ref}}^{2}(\%/{}^{\circ}C)$$

To discuss the effects of *δ* on the luminophore concentration, it is non-dimensionalized as follows:

(7) $$\mathrm{norm}\delta =\frac{\delta -\delta_{\text{min}}}{\delta_{\text{max}}-\delta_{\text{min}}}$$

where *δ*_max_ and *δ*_min_ are the maximum and the minimum temperature dependencies, respectively. Overall, our *δs* showed negative (see Section 3.3). This means that *δ*_min_ is the most temperature dependent and *δ*_max_ the least temperature dependent. Therefore, higher the *normδ* gives less temperature dependent AA-PSP.

### 2.5. Signal Level

The luminescent signal, *I*, is determined by the integration of AA-PSP spectrum from 600 to 700 nm. Based on Liu *et al*., this can be described by the gain of the photo-detector in our spectrometer, *G*, the emission from AA-PSP, *I_AAPSP_*, the excitation in the spectrometer, *I_ex_*, and the measurement setup component, *f_set_* [6]:

(8) $$I=G\;I_{\mathrm{AAPSP}}I_{\mathrm{ex}}f_{\mathrm{set}}$$

In our calibration setup, *G*, *I_ex_*, and *f_set_* were the same for all AA-PSP samples. We non-dimensionalized the luminescent signal by that of AAPSP_00.100_. All luminescent signals were determined at the reference conditions. We call this value as the signal level, *η*, shown in Equation (9):

(9) $$\eta =\frac{I}{I_{\mathrm{AAPSPc}3}}(\%)$$

To discuss the effects of *η* on the luminophore concentration, it is non-dimensionalized as follows:

(10) $$\mathrm{norm}\eta =\frac{\eta -\eta_{\text{min}}}{\eta_{\text{max}}-\eta_{\text{min}}}$$

where *η*_max_ and *η*_min_ are the maximum and the minimum signal levels, respectively.

## 3. Results

### 3.1. AA-PSP Spectrum

**Figure 5.**
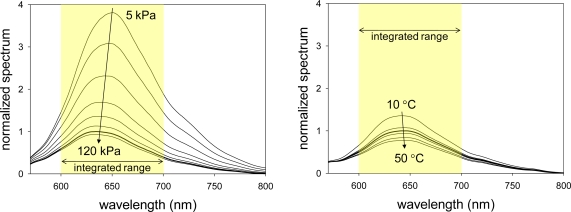
(a) Pressure spectra and (b) temperature spectra of AAPSP_00.100_. Thick line shows the spectrum at reference conditions of 100 kPa and 25 °C.

Figure 5a,b shows luminescent spectra of AAPSP_00.100_ with varying pressures and temperatures, respectively. Spectra were normalized by the luminescent peak at the reference conditions. We can see that, as increasing the pressure, the luminescent spectrum decreased due to oxygen quenching [5].

As the temperature increases, we can see the spectrum decreased due to the thermal quenching [5]. It is noticed that the luminescent peak is shifted from 650 to 635 nm by increasing the pressure from 5 to 120 kPa. For temperature spectra, the peak is shifted from 640 to 645 nm by increasing the temperature from 10 to 50 °C. As described in Section 2.2, we integrated an obtained spectrum from 600 to 700 nm to determine as the luminescent intensity, *I*, for a given pressure and a temperature.

### 3.2. Pressure Calibration

**Figure 6.**
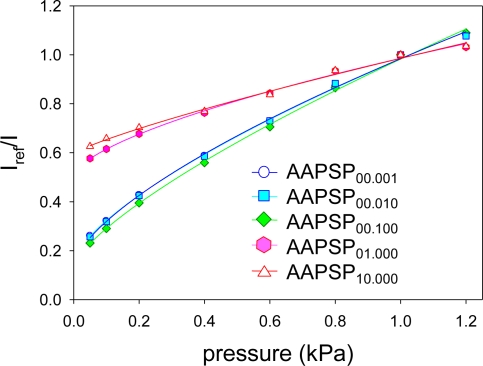
Pressure calibration results.

Figure 6 shows pressure calibrations. Calibration plots were fitted with Equation (2). We can see two groups in calibrations: the luminophore concentration up to 0.1 mM and the concentration higher than 0.1 mM. The former showed steeper calibrations than the latter. This tells us that the former group is more pressure sensitive than the latter.

The pressure sensitivity, *σ*, was determined by using Equation (3) (Table 2). AA-PSP with the luminophore concentration up to 0.1 mM showed *σ* around 60%, while AA-PSP with higher concentration than 0.1 mM showed *σ* around 30%. This tells us that even though the amount of luminophore over 0.1 mM was dissolved in the dipping solution, *σ* did not increase. The decrease in *σ* may be due to the concentration quenching [5].

### 3.3. Temperature Calibration

**Figure 7.**
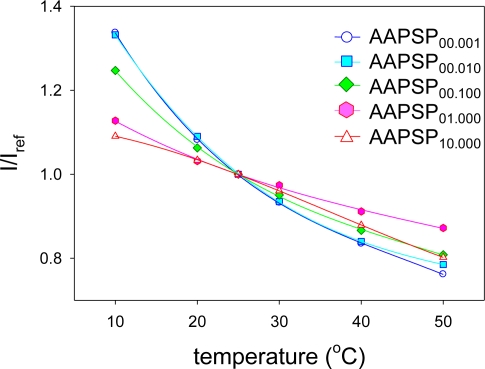
Temperature calibration results.

Figure 7 shows temperature calibrations. Calibration plots were fitted with the third order polynomial described in Equation (5). The calibrations show a monotonic decrease in luminescent signal as the temperature increased. As the concentration decreases, the calibrations become steep. This tells us that the temperature dependency tends to increase as the luminophore concentration decreases.

The temperature dependency, *δ*, was determined from Equation (6) (Table 2). As we increased the luminophore concentration, *δ* decreased. Roughly, *δ* became more than a half by setting the luminophore concentration from 0.001 to 10 mM.

### 3.4. Luminescent Signal

The signal level, *η*, was determined from Equation (9) (Table 2). As we increased the luminophore concentration from 0.001 to 0.1 mM, *η* increased. Even though we increased the concentration more than 0.1 mM, *η* decreased roughly by a half. This may be due to concentration quenching [5]. There is an optimum concentration to maximize *η*. The maximum *η* was obtained from AAPSP_00.100_, whose luminophore concentration was 0.1 mM. Compared to the minimum *η*, there was 3.6 times greater *η* was obtained from AAPSP_c3_.

## 4. Discussion

### 4.1. Optimum Luminophore Concentration for Dipping Deposition Method

**Figure 8.**
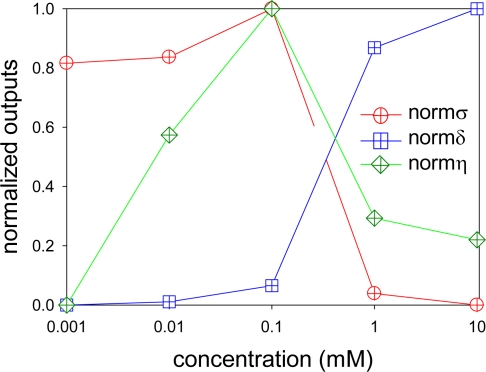
Normalized outputs of AA-PSP. The pressure sensitivity is shown as *normσ*, the temperature dependency as *normδ*, and the signal level as *normη*, respectively.

To consider an optimum condition of dipping deposition method, we plotted normalized quantities of, *σ, δ*, and *η*. These are shown in Figure 8 as *normσ*, *normδ*, and *normη*, respectively. As a pressure sensor, we need *δ* to be zero or close to zero. At the same time, we need higher *σ* as well as higher *η* to give higher luminescent output for a given pressure. These conditions match when all the normalized outputs in Figure 8 are unity. When we look at Figure 8, there is no such a case. The *normσ* and *normη* have similar trend but *normδ* is basically the opposite. The *normσ* and *normη* showed optimum at the luminophore concentration of 0.1 mM. At the concentration higher than 0.1 mM, both *normσ* and *normη* did not increase. On the other hand, *normδ* increased as increasing the luminophore concentration. The change in *normδ* is relatively small from 0.001 to 0.1 mM, but a larger change from 0.1 to 10 mM.

To determine an optimum condition of dipping deposition method, we introduce weight coefficients, *α_σ_ α_δ_*, and *α_η_*. A sum of these coefficients is unity. We arbitrarily determine the importance of these coefficients depending on our sensing purposes. By using weight coefficients, we determine an optimum value, *n_opt_*, as follows:

(11) $$n_{\mathrm{opt}}=\alpha_{\sigma }\cdot \mathrm{norm}\sigma +\alpha_{\delta }\cdot \mathrm{norm}\delta +\alpha_{\eta }\cdot \mathrm{norm}\eta$$

Equation (11) tells us that the maximum *n_opt_* gives an optimum condition of dipping deposition method for given weight coefficients. If we need to maximize the pressure sensitivity but neglect the other factors, we can set *α_σ_* as unity and others as zero. This condition is labeled as conditions *1, and *n_opt_* are listed in Table 3. In this weight condition, AAPSP_00.100_ gives an optimum. If we design an AA-PSP such that all three outputs are equally important, we set *α_σ_, α_δ_*, and *α_η_* as 1/3. The value *n_opt_* was listed in Table 3 as condition *2.

An optimum AA-PSP can be obtained from AAPSP_00.100_. If we design an AA-PSP to minimize the temperature dependency and other outputs are equally important, we can choose *α_σ_* is 0.1, *α_δ_* is 0.8 and *α_η_* is 0.1, respectively. In this case, *n_opt_* is listed in Table 3 as condition *3. An optimum AA-PSP can be obtained from AAPSP_10.000_. By introducing *n_opt_*, we can design an AA-PSP for our sensing purposes related to the luminophore concentration.

### 4.2. Repeatability

**Figure 9.**
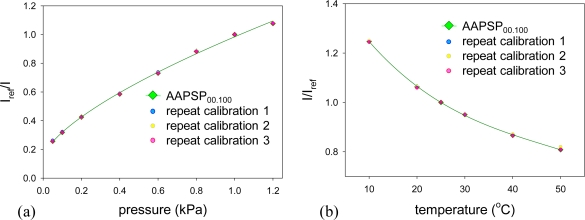
(a) Repeated pressure- and (b) repeated temperature-calibrations of AAPSP_00.100_.

Figures 9a,b show repeated pressure- and temperature-calibrations of AAPSP_00.100_. Pressures and temperatures were increased from lower to higher values for obtaining the original pressure and temperature calibrations of AAPSP_00.100_. Repeat calibrations 1 and 3 decreased pressures and temperatures, and repeat calibration 2 increases these values, respectively. Calibrations were done as a repeating cycle. As one can see, almost all the calibration points at a given pressure and temperature overlap. The maximum error based on the standard deviation was ±0.3% for the pressure calibration and ±0.6% for the temperature calibration, respectively.

**Figure 10.**
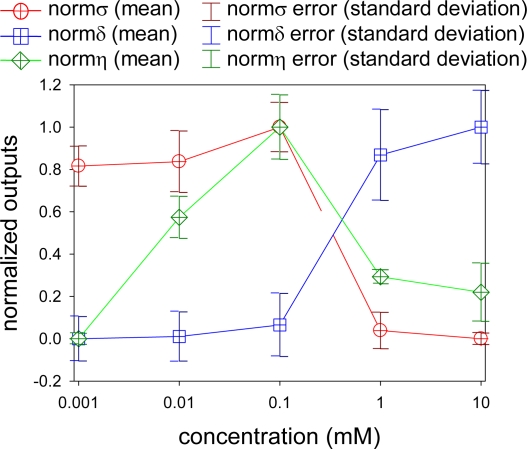
Normalized outputs of AA-PSP with standard deviations as errors bars.

Figure 10 shows normalized outputs with error bars. We prepared three samples for each luminophore concentration and for each sample, normalized outputs were determined. The mean values are shown with their standard deviations as error bars. The results discuss the repeatability of AA-PSP preparation. There is relatively a large error at the luminophore concentration of 1 mM, which gave ±21% error of *normδ*, while, most of the errors are within ± 10%. Causal factors for this error may be the temperature of the dipping solution, dipping duration of the anodized coating, and the calibration fitting error. The former two factors can be minimized by preparing the sample at the same time. However, to discuss the repeatability of AA-PSP preparation, each sample was dipped separately. Small variations of these factors may cause the error. However, as shown in Figures 9a,b, a provided AA-PSP showed a good repeatability in cycling of the pressures and the temperatures. For the real application, a provided AA-PSP should be calibrated to minimize the effects on dipping deposition factors. The calibration fitting error is related to the determination of calibration constants, which is directly related to the normalized outputs. This error can be minimized by increasing calibration points.

## 4. Conclusions

Optimization of anodized-aluminum pressure-sensitive paint (AA-PSP) by controlling the luminophore concentration in the dipping deposition method was studied. It was varied from 0.001 to 10 mM. The relationship between the concentration and AA-PSP characteristics was shown. Characterizations include the pressure sensitivity, *σ*, the temperature dependency, *δ*, and the signal level, *η*. It was found that an optimum concentration exists to increase *σ* and *η*. When we increase the concentration, *δ* decreased. Roughly speaking, *σ* and *η* showed similar trends with the concentration, while *δ* showed the opposite one. By introducing weight coefficients, we could determine an optimum luminophore concentration to provide an optimized AA-PSP for our sensing purposes.

## Figures and Tables

**Table 1.** Dipping conditions of AA-PSP. Dipping solvent was dichloromethane. Anodized-aluminum coatings were dipped at room temperature for one hour.

**Sample ID**	**Luminophore Concentration (mM)**
AAPSP_00.001_	0.001
AAPSP_00.010_	0.01
AAPSP_00.100_	0.1
AAPSP_01.000_	1
AAPSP_10.000_	10

**Table 2.** Summary of AA-PSP characterization results.

**Sample ID**	**Pressure Sensitivity *σ* (%)**	**Temperature Dependency *δ* (%/°C)**	**Signal Level *η* (%)**
AAPSP_00.001_	57	−1.44	27.5
AAPSP_00.010_	58	−1.43	69.1
AAPSP_00.100_	62	−1.38	100.0
AAPSP_01.000_	33	−0.73	48.7
AAPSP_10.000_	31	−0.62	43.4

**Table 3.** Optimum value, *n_opt_*, determined from weight coefficients, *α_σ_*, *α_δ_*, and *α_η_* for given luminophore concentration. Condition *1: *α_σ_* = 1 and others are zero. Condition *2: *α_σ_* = *α_δ_* = *α_η_* = 1/3. Condition *3: *α_δ_* = 0.8, and *α_σ_* = *α_η_* = 0.1.

**Sample ID**	*n_opt_* *1	*n_opt_* *2	*n_opt_* *3
AAPSP_00.001_	0.8160	0.2720	0.0816
AAPSP_00.010_	0.8368	0.4738	0.1497
AAPSP_00.100_	1.0000	0.6884	0.2522
AAPSP_01.000_	0.0389	0.4001	0.7278
AAPSP_10.000_	0.0000	0.4066	0.8220
